# Insights into Galvanic Corrosion Behavior of Ti-Cu Dissimilar Joint: Effect of Microstructure and Volta Potential

**DOI:** 10.3390/ma11101820

**Published:** 2018-09-25

**Authors:** Ehsan Rahimi, Ali Rafsanjani-Abbasi, Amin Imani, Saman Hosseinpour, Ali Davoodi

**Affiliations:** 1 Materials and Metallurgical Engineering Department, Faculty of Engineering, Ferdowsi University of Mashhad, Mashhad 9177948974, Iran; rahimi.ehn@gmail.com (E.R.); ali.rafsanjani@gmail.com (A.R.-A.); 2 Polytechnic Department of Engineering and Architecture, University of Udine, Via del Cotonificio 108, 33100 Udine, Italy; 3 Materials and Polymers Engineering Department, Hakim Sabzevari University, Sabzevar 9617976487, Iran; aminimani88@gmail.com; 4 Institute of Particle Technology (LFG), Friedrich-Alexander-Universität-Erlangen-Nürnberg (FAU), Cauerstraße 4, 91058 Erlangen, Germany

**Keywords:** Ti-Cu dissimilar welding, microstructure, electrochemical measurements, galvanic corrosion, SKPFM

## Abstract

The effect of microstructure on corrosion behavior of a solid-state explosion welded Ti-Cu bimetal is investigated by means of alternating current-direct current (AC-DC) electrochemical measurements, optical microscopy, scanning electron microscopy, and scanning Kelvin probe force microscopy (SKPFM). The results indicate that the titanium regions in the welding interface, local melted zone (LMZ), and LMZ-Cu interface are potential sites for initiation of corrosion attacks. SKPFM mapping clearly shows that before exposure of the sample to a 3.5% NaCl corrosive solution and at the beginning of the exposure, the Cu side of the bimetal has a higher Volta potential in comparison to that of the Ti region, and thus acts as a cathode. Electrochemical measurements also confirm that titanium acts as an anode and copper as a cathode, in the first moments of immersion, in accordance with macroscopic observations and SKPFM results. However, by growing a passive layer of titanium oxide and titanium hydroxide on the Ti side after about 1 h exposure to the corrosive medium, the titanium side becomes nobler and the polarity arrangement in the galvanic couple reverses.

## 1. Introduction

Cladding by explosion welding is a specific type of solid-state bonding method for fabrication of bimetals and multilayer composites, consisting of dissimilar metals or alloys [1], where mismatching chemical and physical properties make it difficult to join them using conventional fusion welding methods. In explosion welding, controlled detonation of explosives forces two or more metals together at high pressures and high temperatures [2]. Steel, aluminum, titanium, nickel, copper, and metallic glass, among many others, are materials that could be effectively joined using explosion welding [3].

Ti-Cu bimetal cladding is a good example, in which the unique properties of its constituents (i.e., Cu and Ti) are combined using explosion welding. Copper is a non-magnetic metal with a wide range of applications, owing to its high electrical and thermal conductivities and good mechanical workability, which is used as pure metal or as alloy [4]. Despite its good resistance against uniform corrosion, copper is susceptible to pitting and localized corrosion in chloride-containing marine environments, which cause a non-uniform localized attack of copper items in offshore installation [4]. Controlling the localized corrosion of copper in severe environments such as desalination facilities, heat exchangers, plumbing, transformers or bearings in the oil industry, and copper corrosion due to electrolyte formation on electronic components or local chloride accumulation in building materials (or atmospheric corrosion) has attracted a lot of attention [5,6,7,8,9]. Titanium, because of its higher corrosion resistance and better mechanical properties in comparison with copper, has proven to be a good candidate to be joined with copper for better corrosion performance [10]. The superior corrosion resistance of Ti compared to Cu is related to the chemically unreactive, condensed and adherent oxide films formed on its surface [10,11]. In fact, Ti-Cu bimetals, which combine the properties of both Ti and Cu, meet multiple necessities in terms of high thermal strength, good corrosion resistance, and high thermal and electrical conductivities for specific applications [12,13,14,15]. As a result, Ti-Cu cladding, prepared by explosion welding, is used in many industries, including the aerospace, automotive, electronic, electrochemical, and metallurgical industries [3,15,16]. 

Relevant basic parameters in explosion welding include flyer velocity, collision point velocity and collision angle [17]. Proper selection of these parameters leads to a strong metallurgical bond between the two metals at their contact point [3]. In the process of explosion welding, however, localized high temperatures are normally generated at the weld interface [18], which can exert a detrimental effect on the performance of the bimetal in application. Also, a jet of materials is formed between metal plates, which partially removes the surface of both plates and causes the formation of waves at their interface [19]. The wavy interface often produces a better welding characteristic in comparison with flat-melted bonds [18]. Therefore, the large and wavy contact area in bimetals often results in good tensile and shear strengths [17]. The mechanism of wave formation and the chemical composition of the interface in explosive welding have been extensively studied [18,19,20]. However, in the course of explosion welding, the considerable extra kinetic energy of the colliding plates may also cause localized melting of metallic plates near the interface, which leads to the formation of intermetallic compounds and reduction of the welding quality at the interface [21]. In such cases, the corrosion resistance of the joint is often compromised [22]. 

From the viewpoint of corrosion, in explosion welding, the subsidiary precipitates that are created during fusion and solidification corrode severely and modify the continuity of the joints [14]. Acarer investigated the corrosion behavior of Al-Cu bimetal produced by explosive welding. It was observed that galvanic corrosion on the aluminum side of the bimetal was more than that on the Cu side [2]. Mudali et al. studied the corrosion performance of titanium-304L SS bonding using two different methods, i.e., friction welding and explosive joining in boiling nitric acid media. Their observations showed that the friction-welded joint undergoes severe corrosion attacks with trench formation at the interface, while the explosive-welded joint has severe corrosion attack on the 304L SS with selective attacks in the vortex or eddy region of the welded interface [16]. Many investigations have shown considerable grain deformation and presence of adiabatic shear bands in the base metal adjacent to the interface due to the local high temperature and collision. These shear bands are found to be potential sites of corrosion in bimetals [1,16,23,24,25]. 

Herein, significant attention is paid to the characterization and evaluation of the microstructure and corrosion behavior of Ti-Cu cladding sheet produced by explosive welding. To achieve this goal, analytical tools such as optical microscopy (OM), scanning electron microscopy (SEM), atomic force microscopy (AFM), and scanning Kelvin probe microscopy (SKPFM) are employed to provide direct and indirect evidence on the preferential corrosion sites at the boundary between the two metals. Also, quantitative information is obtained from electrochemical measurements and the correlation between numerical and qualitative results is rigorously investigated.

## 2. Materials and Methods 

### 2.1. Materials and Preparation

**Figure 1:**
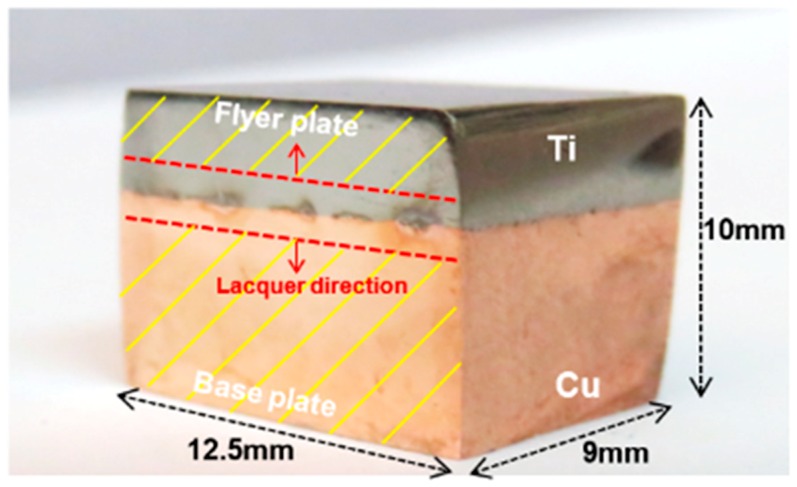
Photograph image of Ti-Cu bimetal sample for experimental measurements.

An industrial Ti-Cu cladded plate from a petrochemical plant produced by explosive welding was used in this study. A Ti sheet with a thickness of 3 mm and a copper sheet with a thickness of 27 mm were used as flyer and base plate, respectively. The sample was received in the form of a large plate and was cut into cubes with a surface area of 1.25 cm^2^ for microstructure characterizations, electrochemical measurements, and AFM/SKPFM evaluations, as shown in Figure 1.

### 2.2. Metallographic Studies and Hardness Measurement

Specimens for microstructure analysis were grounded using successive grades of SiC papers up to 3000 grit and were polished with alumina-impregnated cloth (according to ASTM E3-11) [26]. Microstructure examination was carried out using a digital camera-assisted optical microscope and a scanning electron microscope (models: LEO 1450 VP, Zeiss, Oberkochen, Germany and Phenom ProX, Phenom-World BV, Eindhoven, Netherland, respectively) equipped with an energy dispersive spectroscopy (EDS) analysis system. For optical studies, the welded sample was etched for 15 s in Kroll’s etchant (98 mL H_2_O + 4 mL HNO_3_ + 2 mL HF), washed with ethanol and finally dried with hot air. Vickers micro-hardness test was implemented using a Buehler micro-hardness machine (load = 200 g, dwell time = 20 s) on a longitudinal section of the weld. For a better accuracy, all the micro-hardness measurements were performed at least three times to ensure the reproducibility of the hardness results.

To detect the distribution of phases and the degree of their crystallinity, X-ray diffraction (XRD) analysis was performed on different regions of the Cu-Ti sample using an Explorer GNR. The X’Pert HighScore software was used for evaluation of the XRD data.

### 2.3. Electrochemical Measurements

Individual specimens of pure Cu and pure Ti were cut from Ti-Cu bimetal sheet, as working electrodes. In addition, for investigation of galvanic behavior between the two metals in joining interface, as is shown in Figure 1, lacquer was applied to both sides of Ti-Cu joint metals, leaving an equal distance of 1 mm from the center of the welded interface uncovered. The surface area of the uncovered region for galvanic measurements was 2 mm × 12.5 mm (0.25 cm^2^) with the Ti-Cu surface ratio of 1:1. Electrodes were separately cold-mounted in epoxy resin with a copper wire connection at the end. Prior to each test, electrodes were prepared by wet grinding up to 3000 grit SiC paper, using ethanol as a lubricant. All electrochemical measurements were carried out in a 3.5 wt.% NaCl solution (pH = 6.7), open to the air at 25 ± 1 °C with a conductivity of 45 ± 2 mS/cm in 500 mL Pyrex glass cell. Electrochemical tests were repeated three times and the results of three replicate measurements were averaged. 

The potentiodynamic polarization (PDP) measurements were carried out at a scan rate of 1 mV·s^−1^ from cathodic to anodic potentials. Electrochemical impedance spectroscopy (EIS) measurements were performed in a frequency range of 10 MHz to 30 kHz by applying a sinusoidal excitation signal of ±10 mV. Also, galvanic measurement of Ti-Cu couple was carried out in 3.5 wt.% NaCl solution for the investigation of the polarity reversal time. All electrochemical measurements were performed with a Gill AC potentiostat (ACM instrument, Cumbria, UK) with a saturated calomel electrode (ACM instrument, Cumbria, UK) (SCE, +245 vs. SHE) and a platinum foil as the reference and counter electrode, respectively.

### 2.4. Volta Potential Measurement by SKPFM

SKPFM measurements were performed with a commercial Solver Next AFM instrument (from NT-MDT Spectrum Instruments, Moscow, Russia) on mirror-like polished specimens. All measurements were performed in air at ambient temperature with a relative humidity of 35 ± 2% using a doped silicon pyramid AFM tip. The surface potential analysis was carried out by employing the dual-scan mode. In the first scan, topography data was recorded using the dynamic (also known as tapping) mode. In the next line scan, potential distribution was evaluated while the tip was lifted up about 70 nm (depending on the surface roughness), following the topography of the same line. The dual scan mode eliminates the effects of topographic features on the potential mapping [27,28]. Volta potential distribution in the scanned area was obtained with a pixel resolution of 512 × 512 and scan frequency rate of 0.3 Hz. Generally, Volta potential is regarded as a measure of the relative nobility of materials [25,29,30]. In this study, we employed SKPFM specifically to assess the potentially localized corrosion initiation sites at the weld interface and melted zones close to it. The SKPFM maps were compared with the SEM and OM images to evaluate the effect of surface constituents and microstructure on the initiation of localized corrosion at and close to the joint interface.

## 3. Results

### 3.1. Microstructural Investigation

**Figure 2:**
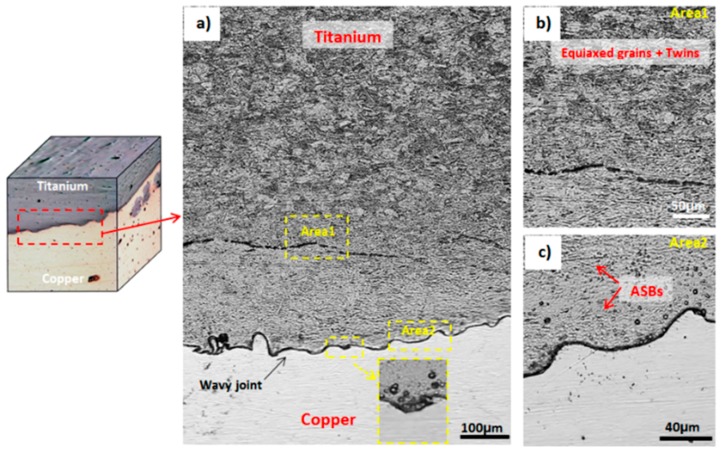
Optical microscopy images showing (**a**) titanium and copper near weld interface, (**b**) titanium microstructure with equiaxed grains and twins, (**c**) adiabatic shear bands (ASBs) and the wavy interface formed between Ti-Cu bimetal joint.

Figure 2 shows the typical wavy morphology of the interface of the cross-section of the explosion-welded Ti-Cu sample. As is depicted in this figure, the deformation of the cladding Ti plate is inhomogeneous. In fact, on the titanium side, the grains are quite equiaxed and a high number of twin bands can be observed (Figure 2a,b), which is similar to observations by Gloc et al. [23]. Adiabatic shear bands (ASB) near the interface, which are due to the local high temperature and high adiabatic shear sensitivity of titanium at high strain rates, are also observable, which is similar to previous studies by Gloc et al. [23] and Song et al. [24]. These shear bands are indicated with arrows in Figure 2c. In general, ASBs consist of fine and small equiaxed grains comprising a large number of twin bands which appear inclined at about 45° to the explosive direction and near the interface [22,31,32]. 

**Figure 3:**
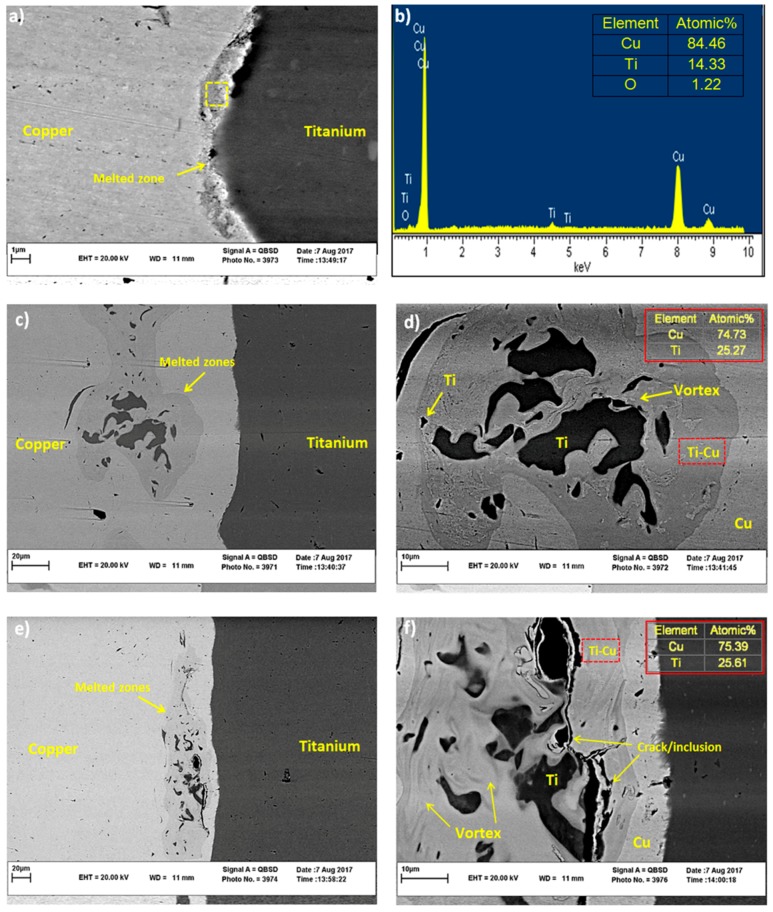
SEM images of (**a**,**b**) the welded interface with EDS analysis results, (**c**,**e**) LMZs, (**d**,**f**) greater magnification and EDS results of Ti-Cu phase at LMZs corresponding to (**c**,**e**) along with Ti particles, vortex, and inclusion/crack.

The inset in Figure 2a indicates the vortex at the Ti-Cu interface. According to [14,24,33,34], both welded materials participate in vortex creation. However, as the melting point of copper is lower than that of titanium, the local melted zone (LMZ) is formed mainly on the copper side of the binary metal. Therefore, in our detailed SEM investigations, we mainly focus on the weld interface and LMZs on the copper side. Figure 3a,b show SEM image and EDS result of LMZ at the weld interface. The region where the EDS result is obtained is marked with a dotted box. The LMZ area can be created due to the high level of energy dissipation in Ti-Cu samples, which is accompanied with atomic diffusion between dissimilar matrixes [12,20]. The LMZ width at the interface of Ti-Cu is small (about 1µm) in comparison with that in other works in this field [20,22,35], possibly due to the higher heat conductivity of copper [14] than the steel used in those studies. The EDS result in Figure 3b indicates a non-equal contribution of Cu and Ti in the LMZ; the amount of Cu in LMZ is much more than Ti. In fact, the difference in melting points of Ti and Cu plays an important role in determining the chemical composition of the melted zone, as was explained in [12,18]. 

Figure 3c,e shows different LMZs on Cu side, along with high magnification images of these zones (Figure 3d,f). Three regions can be clearly observed in LMZs in Figure 3d,f, including Ti-Cu intermetallic phase, pure Ti regions, and impurities. These zones are attributed to high explosive power and ejection process between flyer and base plate after the explosion [14]. Rapid solidification and further cooling of the Ti-Cu sample are responsible for the formation of micro-cracks within some of the brittle impurities, as marked in Figure 3f [24].

**Figure 4:**
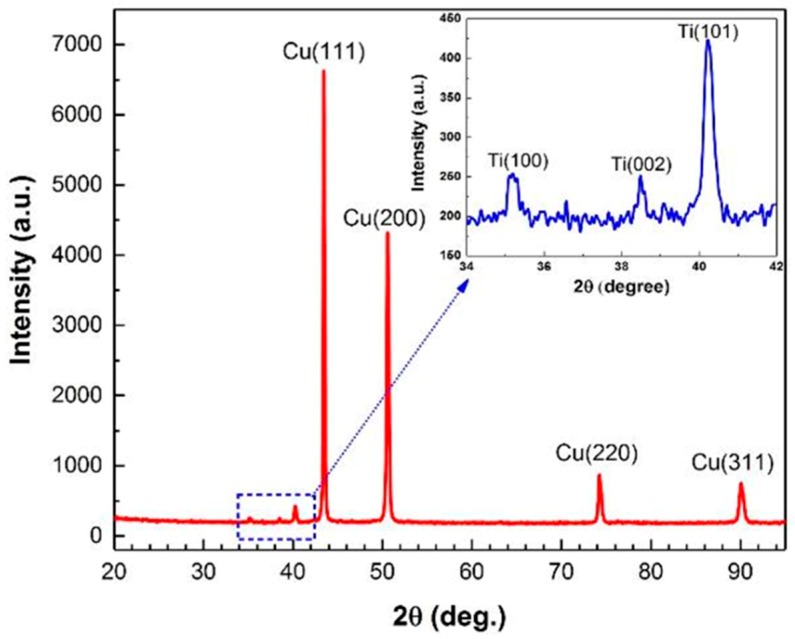
XRD pattern for Ti-Cu bimetal in the adjacent of weld interface.

According to the EDS analysis results in Figure 3b, and based on the Ti-Cu binary alloy phase diagram, the melting regions in Figure 3 are expected to be composed of Ti_2_Cu_3_, TiCu_2_ and TiCu_4_ phases with different distributions in their atomic proportions [36]. Elrefaey and Tillmann suggested that a eutectic mixture of TiCu_2_ and TiCu_4_ will form in the middle of the joint [37]. Also, Saboktakin et al. discussed the formation of Ti_2_Cu_3_ and TiCu_4_ in transition layers between Ti and Cu metals in the roll bonding process of titanium clad steel with the copper interlayer [38]. A similar investigation by Guoyin et al. on the microstructure and mechanical behavior of explosive-welded Ti-Cu showed that the TiCu phase in LMZ contains 50.19% Ti and 49.81% Cu [12], similar to their binary alloy phase diagram. However, XRD analysis of the Ti-Cu weld interface in this study exhibits only Ti and Cu peaks, as shown in Figure 4. The absence of intermetallic peaks in the XRD pattern is thus attributed to the formation of either amorphous intermetallics or a low amount of these intermetallics, below the detection limit of our XRD, in accordance with what Rajani et al. found for Inconel 625/plain carbon steel bimetal plate [20]. 

### 3.2. Micro-Hardness Test

**Figure 5:**
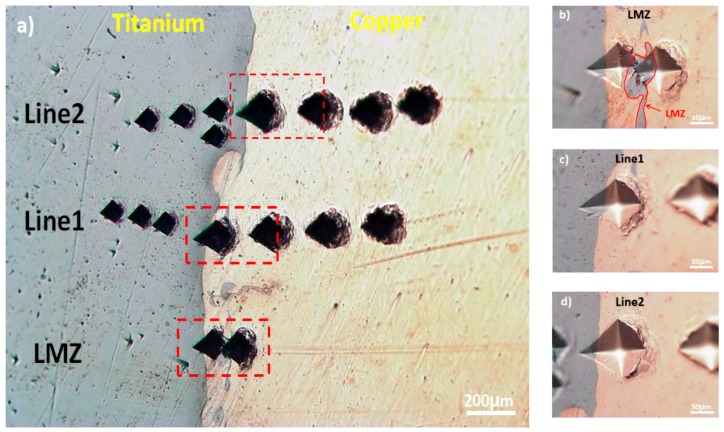

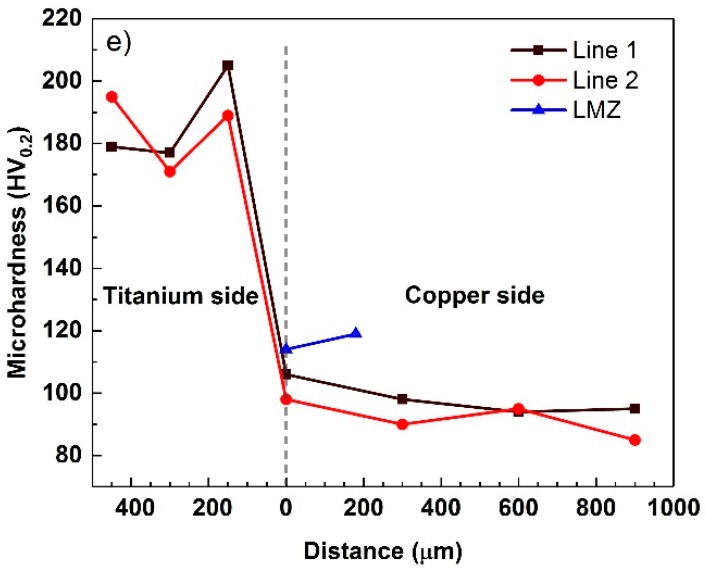
(**a**) Optical micrograph of microhardness distribution in the Ti-Cu bimetal, (**b**) on LMZ, (**c**,**d**) in the vicinity of the interface, and (**e**) micro-hardness profiles along a welded interface and LMZ.

The optical image of hardness indentation and micro-hardness profiles across the explosive-welded Ti-Cu bimetal are shown in Figure 5a,e, respectively. The pure titanium and pure copper regions at locations far from the Ti-Cu interface exhibit the average micro-hardness of approximately 185 HV_0.2_ and 90 HV_0.2_, respectively. Similar studies by Kahraman et al. [17] showed that the highest hardness for pure Ti and Cu is obtained in the vicinity of the welded interface, due to the highest collision speed at the interface during explosive welding. It can be observed from Figure 5e that the maximum hardness is indeed achieved on the Ti side close to the joint interface, which reflects the more severe plastic deformation on the Ti side of the weld. As is depicted in Figure 5a,b, the presence of Cu particles dispersed or melted throughout the interface promotes a softening effect in this zone [19]. Due to the presence of pure Ti particles and Ti-Cu intermetallic phases, the hardness of the LMZ (117 ± 2 HV_0.2_) was a bit more than the hardness at the interface (102 ± 2 HV_0.2_).

### 3.3. Corrosion Behavior

**Figure 6:**
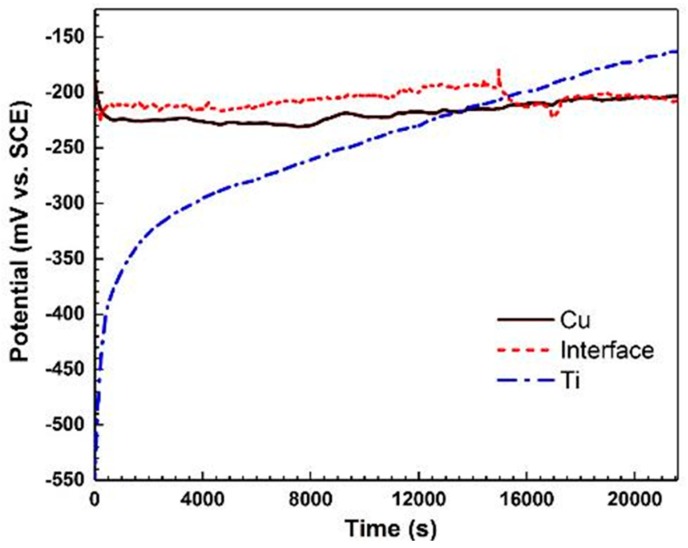
Open circuit potential versus time for Ti, Cu and weld interface in 3.5 wt.% NaCl solution for 6 h.

The open circuit potential (OCP) values for three regions on the titanium side, the weld interface and the copper side in 3.5 wt.% NaCl solution are shown in Figure 6. It is evident that the Ti region shows an increase in potential value with exposure time, which is in accordance with the detailed SKPFM studies on a similar system [25]. This increase in potential is attributed to the formation of a surface passive layer on the Ti side of the bimetal (i.e., growth of a thin TiO_2_ passive layer) [10,11,39]. According to [40,41], this passive film consists of an outer hydroxide layer, due to the reaction with water molecules, and an inner oxide layer, as a result of metal oxidation. Both inner and outer layers contribute to the high corrosion resistance behavior of the Ti side of the bimetal. In contrast to the Ti side of the joint, the Cu region shows only a minor change in potential value during exposure to the corrosive solution. The variations of the potential of the Cu side is a function of two counteracting processes, i.e., (a) formation of a protective film of cuprous oxide (Cu_2_O), and (b) adsorption of a large amount of Cl^−^ on the cuprous oxide matrix and formation of Cu_2_(OH)_3_Cl, which leads to a slight reduction of the corrosion potential [9,42]. In fact, corrosion of copper in non-deoxygenated seawater solution involves the reduction of oxygen in the cathodic reaction. Cathodic and anodic reactions in this case can be expressed as [9,43]:

O_2_ + 2H_2_O + 4e^−^ → 4OH^−^ (1)

Cu → Cu^+^ + e^−^ (2)

followed by formation of the cuprous complex and then copper oxide formation [6]:

Cu^+^ + 2Cl^−^ → CuCl_2_^−^ (3)

2CuCl_2_^−^ + 2OH^−^ → Cu_2_O + H_2_O + 4Cl^−^ (4)

which is further oxidized to the atacamite or the isomorphous phase paratacamite (Cu_2_(OH)_3_Cl), which has minor protective characteristics [5,6]:

2Cu_2_O + O_2_ + 2Cl^−^ + 4H_2_O → 2Cu_2_(OH)_3_Cl + 2OH^−^ (5)

The OCP of the weld interface experiences a decrease in the potential of −205 mV vs. SCE after almost 4.5 h exposure to the corrosive environment and finally reaches a steady-state condition. These changes in the OCP of the weld interface can be attributed to the galvanic coupling effect, local variations in material composition, and microstructure in the welded interface, especially in LMZs [44]. The large difference in potential between the two metals after ca. 6 h exposure to NaCl solution clearly indicates the possibility of a galvanic coupling at the joint interface. 

**Figure 7:**
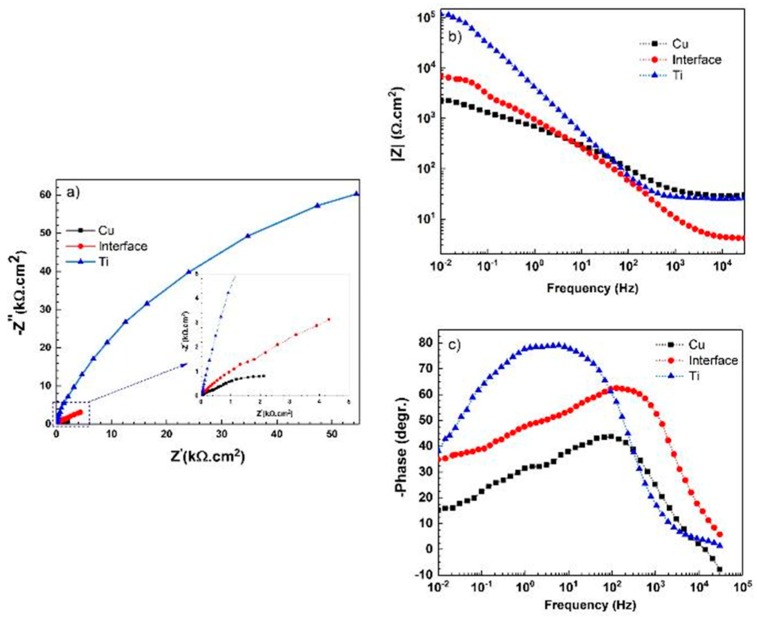
(**a**) Nyquist, (**b**) Bode |Z| and (**c**) Bode-phase diagrams resulting from electrochemical impedance spectroscopy of Ti, Cu and weld interface in 3.5 wt.% NaCl solution; 10 mV amplitude around E_ocp_ with a frequency range of 30 kHz–10 MHz.

After reaching a steady state in OCP values, EIS and polarization experiments were conducted in all three regions (i.e., on the Cu side, on the Ti side, and at the interface). Experimental results of EIS measurements, including Nyquist, Bode |Z| and Bode-phase, are shown in Figure 7. Figure 7a shows that the corrosion resistance of the three regions is in the order: Cu > interface > Ti. According to Figure 7b, the corrosion resistance of the welded region is between that of the copper and the titanium, which is also in agreement with the results in Figure 7a,c. 

Figure 7c depicts that Ti has a one-time constant at low and medium frequencies, in contrast to the Cu side and the weld interface which show two-time constants. The phase value for the Ti side approaches 77°, which is due to the formation of a dense oxide film of TiO_2_ with a high dielectric constant in this corrosive media [10,39,45]. The two-time constants for Cu and weld interface are related to the formation of oxide or corrosion layer at higher frequencies and the corroding interface at lower frequencies. It is expected that LMZs play a key role in the corrosion performance of the welded interface. In the Nyquist diagram, the interception of the curve with the real axis at the higher frequency is associated with the solution resistance (R_s_), and the interception at the lower frequency is attributed to the charge transfer resistance (R_ct_). For the Ti region, with a compact passive layer, the R_ct_ value is equivalent to the film resistance (R_f_), which is an important parameter in the assessment of corrosion performance. In general, the titanium region exhibits a higher corrosion resistance than that of other regions. 

**Figure 8:**
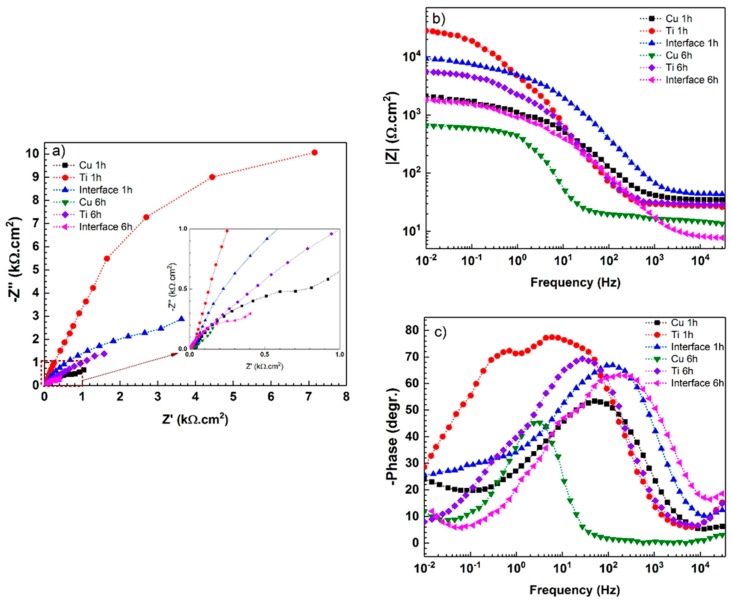
(**a**) Nyquist, (**b**) Bode |Z| and (**c**) Bode-phase diagrams resulting from electrochemical impedance spectroscopy of Ti, Cu and weld interface in 3.5 wt.% NaCl solution after 1 h and 6 h immersion around E_ocp_.

To investigate the different surface properties of the three regions after longer immersion times (1 h and 6 h), EIS measurements were performed again and the results are provided in Figure 8.

**Figure 9:**
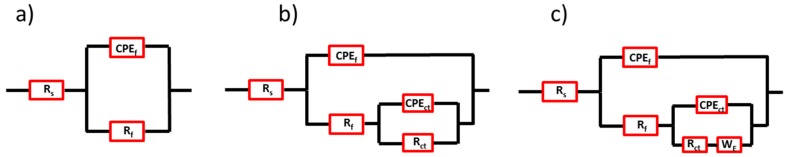
Equivalent circuits for EIS data of Ti, Cu and weld interface immersed in 3.5 wt.% NaCl solution: (**a**) Ti for 0 h; (**b**) Cu and weld interface for 0 h, Ti for 1 h and 6 h; (**c**) Cu and weld interface for 1 h and 6 h.

EIS results can be analyzed with the electrical equivalent circuits, as shown in Figure 9. In these circuits, CPE_dl_ and CPE_f_ are the constant phase elements of double-layer and film (the passive layer or the corrosion products), respectively. The extracted result from the fitted data is presented in Table 1. The impedance of CPE is defined as the following equation [46]:

(6) $$\;Z_{\mathrm{CPE}}=\frac{1}{Y_{0}\;{\left(j\omega \right)}^{n}}\;$$

where Y_0_ is the modulus, j is an imaginary unit, ω is the angular frequency, and n is the CPE exponent (−1 ≤ n ≤ 1). CPE describes an ideal inductor for n = −1, an ideal resistor for n = 0, and an ideal capacitor for n = 1 [45]. The “n” value in CPE is an indication of surface roughness; the lower value of it indicates the rougher or more heterogeneous surface. For the titanium side, the n_1_ = 0.89 value is a characteristic of native barrier oxide film with a slight deviation from the ideal capacitor. For the copper side and weld interface, the “n_2_” value is much lower than that on the titanium side. Therefore, the oxidation of the Cu side, as well as the dissolution of Ti-Cu intermetallic phases in the weld interface, leads to roughening of the surface on the Cu side and at the joint interface [44].

According to Figure 8 and Table 1, after 1 h immersion in 3.5 wt.% NaCl solution, all three regions (i.e., copper side, titanium side, and the weld interface) show different surface properties. It can be observed that the impedance spectra of titanium have been switched from one-time constant to two-time constants in the range of 10^2^ to 10^−1^ Hz (Figure 8c). This behavior is due to the growth of oxide film with a two-layer structure which was developed during the corrosion [45,47]. In this period, R_f_ decreased from 123 to 4.7 kΩ·cm^2^. On the other hand, Nyquist plots of the weld interface and the copper sample show a capacitive loop at high to moderate frequencies. The straight line or Warburg (W) impedance (W is the Warburg diffuse element) at low frequencies can be modeled with the equivalent circuit, as shown in Figure 9c. It can be concluded that more corrosion products (or corrosion attacks) are formed during immersion, and therefore, charge transfer resistance decreases [48,49]. As was mentioned earlier, the value of n_2_, as a parameter for measuring the roughness of the surface and its heterogeneity, is also influenced by immersion time due to more corrosion attacks. As can be observed in Table 1, Ti, Cu and weld interface regions present n values in a range between 0.43 and 0.52. By increasing the immersion time from 1 h to 6 h, the Nyquist and Bode phase plots of three samples show similar changes in surface properties. However, much more severe degradation is observed after 6 h immersion in the corrosive solution. The decrease in EIS parameters, including W, R_f_, R_c_, and n_2_, with exposure time to the corrosive medium clearly demonstrates the gradual degradation of the Ti, Cu, and weld interface regions.

**Figure 10:**
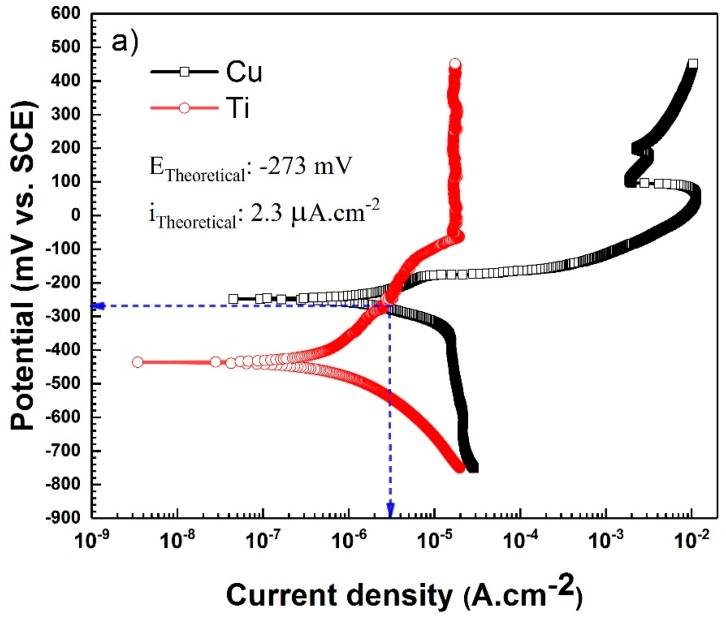

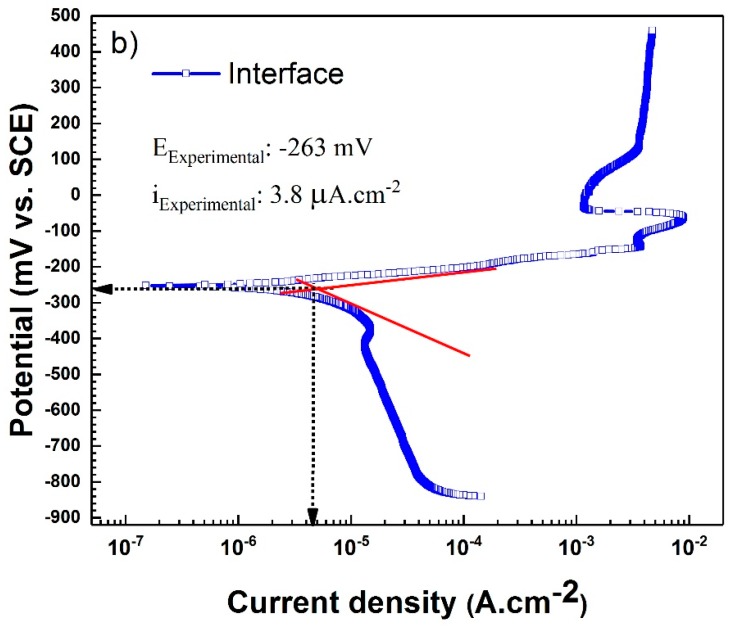
The plots of potentiodynamic polarization in 3.5 wt.% NaCl solution: (**a**) uncoupled Cu and Ti surfaces along with theoretical galvanic current and potential; (**b**) weld interface with 1:1 surface ratio along with experimental galvanic current and potential.

The PDP curves of the three regions on the Ti-Cu bimetal (i.e., titanium, copper, and weld interface) are provided in Figure 10a,b, and their corresponding relevant fitting parameters from Tafel extrapolation are summarized in Table 2. As can be seen, Ti has a lower corrosion current density in comparison with Cu. Furthermore, unlike Cu and the interface regions, which are prone to pitting at increased potentials, titanium shows an outstanding stability with no trace of pitting corrosion behavior in the examined potential ranges. The resistance of titanium to pitting is attributed to the formation of the natural oxide film on titanium and an increase in the corrosion resistance of the oxide layer upon exposure to the corrosive medium [45]. Based on the results presented in Figure 10a, the Ti region shows passivity in the anodic direction, followed by an activation control in cathodic polarization. This non-trivial active-passive transition was previously observed in the PDP curve of Ti in NaCl solution [40,50]. As can be seen in Figure 10a,b, both the Cu region and the interface region exhibit a pseudo-passive behavior in the anodic branch and a diffusion-controlled behavior in the cathodic branch.

Theoretical galvanic potential and galvanic current of uncoupled Ti-Cu electrodes can be obtained using the mixed potential theory, by means of two separate Ti and Cu potentiodynamic polarization curves [46]. The PDP curves in Figure 10 show nearly equal values between the experimentally measured galvanic potential and current values and those obtained theoretically: i_theo_ = 2.3 and i_exp_ = 3.8 µA·cm^−2^; E_theo_ = –273 and E_exp_ = −263 mV vs. SCE. 

**Figure 11:**
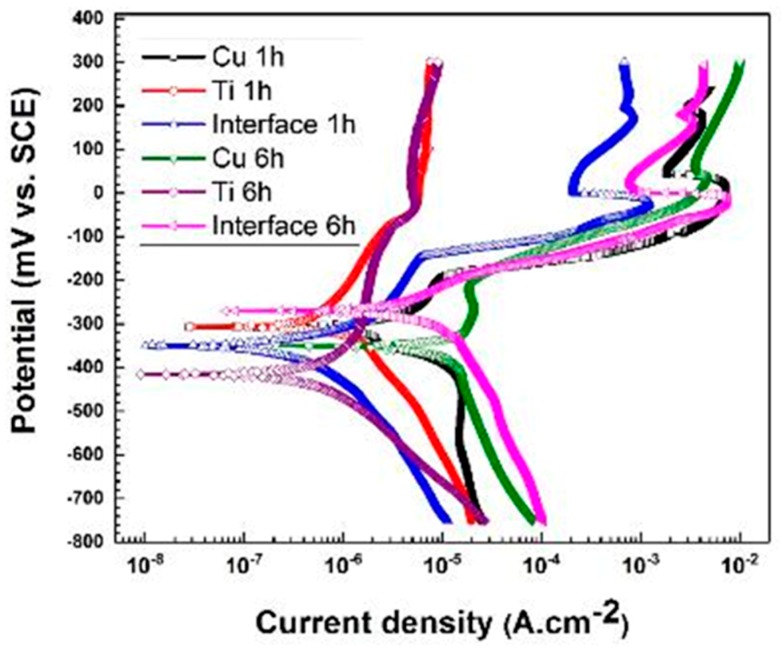
The plots of potentiodynamic polarization of Ti, Cu and weld interface immersed in 3.5 wt.% NaCl solution for 1 and 6 h.

Figure 11 depicts the PDP curves of the Ti, Cu and weld interface regions after immersion in the corrosive solution for two different durations: 1 h and 6 h. It can be observed that the corrosion current density and the corrosion potential for all regions have increased gradually with longer immersion times. As was explained earlier, the increase in the corrosion current density in the Ti region with exposure time is due to the growth of passive film along with localized corrosion [10,45]. Also, more interactions between copper and chloride ions in the NaCl corrosive solution result in a higher corrosion current density and formation of nantokite (CuCl), which usually transforms into paratacamite (Cu_2_(OH)_3_Cl) as a corrosion product [5]. The presence of intermetallic components in the weld interface with different nobility behaviors and especially galvanic coupling effect of Ti-Cu lead to a severe degradation in this region and result in an increase in the corrosion current density on the weld interface region over exposure time in the corrosive solution.

**Figure 12:**
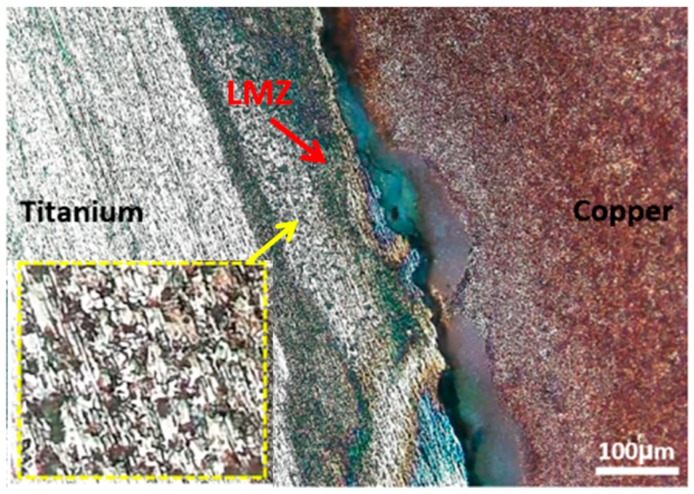
(**a**) Optical micrograph of the galvanic corrosion in the welded zone after PDP test in 3.5 wt.% NaCl solution. The inset shows a greater magnification of the localized corrosion attacks on the Ti side. The red arrow depicts the local melted zone at the joint interface.

To correlate the electrochemical behavior of different regions in the Ti-Cu bimetal to macroscopic events at the joint interface and the areas close to it, optical images were obtained after 0.5 h PDP test, from a large area covering all three regions. As can be seen in Figure 12, some minor localized corrosion occurs on the Ti side of the Ti-Cu bimetal. These localized attack sites could be attributed to the inclusions in pure Ti, which initially act as initiation sites for localized corrosion in the presence of the aggressive ions such as Cl^−^ [10,51]. Within the same exposure period, a more severe corrosion attack is observed on the Cu side of bimetal, which may be counterintuitive, considering the fact that Cu in the more noble metal in the Ti-Cu bimetal. As was described earlier, with thorough AFM-SKPFM studies of the Cu-Ti bimetal [25], the severe corrosion of the copper side is related to the reversal of the polarity (relative nobility) of Cu versus Ti, due to the formation and growth of the oxide layer on the Ti side [9,10,45].

**Figure 13:**
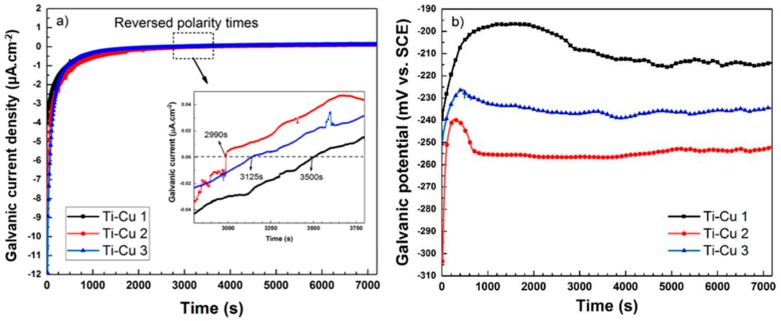
(**a**) Current and (**b**) potential curves of galvanically polarized Ti/Cu couple in 3.5 wt.% NaCl corrosive solution with three repetitions; polarity reversal time or change in the net flow of electrons occurred after approximately 1 h.

To determine the polarity reversal time in the Ti-Cu bimetal, currents and potentials of the galvanic couple are investigated. According to the OCP results in Figure 6, a significant change can be seen in nobility behaviors of the two metals after almost 6 h immersion in 3.5% wt NaCl corrosive solution. In Figure 13a the galvanic current density of the Ti-Ci bimetal in 3.5% wt NaCl solution is plotted for three replicates. Based on these galvanic current curves, negative galvanic current values (below the zero dashed line in the inset) indicate the net flow of electrons from Ti (as anode) to Cu (as cathode) [52,53] in the galvanic couple. Similarly, positive galvanic current values (above the zero dashed line in the inset) reflect the net flow of electrons from Cu (as anode) to Ti (as cathode). The reversal of the polarity occurs at exposure times close to 1 h (indicated by a dotted box in Figure 13a).

**Figure 14:**
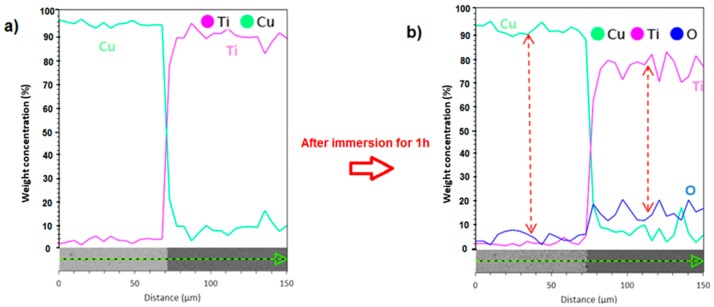
Ti, Cu and O element distribution measured by EDS line analysis (**a**) before and (**b**) after immersion in 3.5 wt.% NaCl corrosive solution for 1 h. The amount of elemental O before exposure was below the detection limit of our EDS analyzer.

According to the galvanic potential curves in Figure 13b, the galvanic potential value for the Ti-Cu bimetal is in the range of −260 mV to −220 mV for the three replicate measurements, which is close to the theoretical and experimental corrosion potentials of the welded interface, *vide supra*. The dramatic increase in the current and potential values at the beginning of galvanic couple measurements indicates a rapid growth of oxide film on Ti side, which increases the corrosion resistance of Ti side and reflects the tendency of changing the Ti behavior from anodic to cathodic [25,52]. The significant growth of oxide film on the Ti side after immersion of the Ti-Cu bimetal in the corrosive solution for 1 h is clearly observable from the EDS line scans in Figure 14. This figure shows the increase of the oxygen content, initially above the measurement threshold, and consequently further increase of the oxygen level on the Ti side of the bimetal. In fact, it has been shown earlier that the hydroxide (TiO_2_·nH_2_O) precipitates cover the Ti surface after exposure to corrosive media [50].

### 3.4. Predication of Volta Potential Measurement and Immersion Test

**Figure 15:**
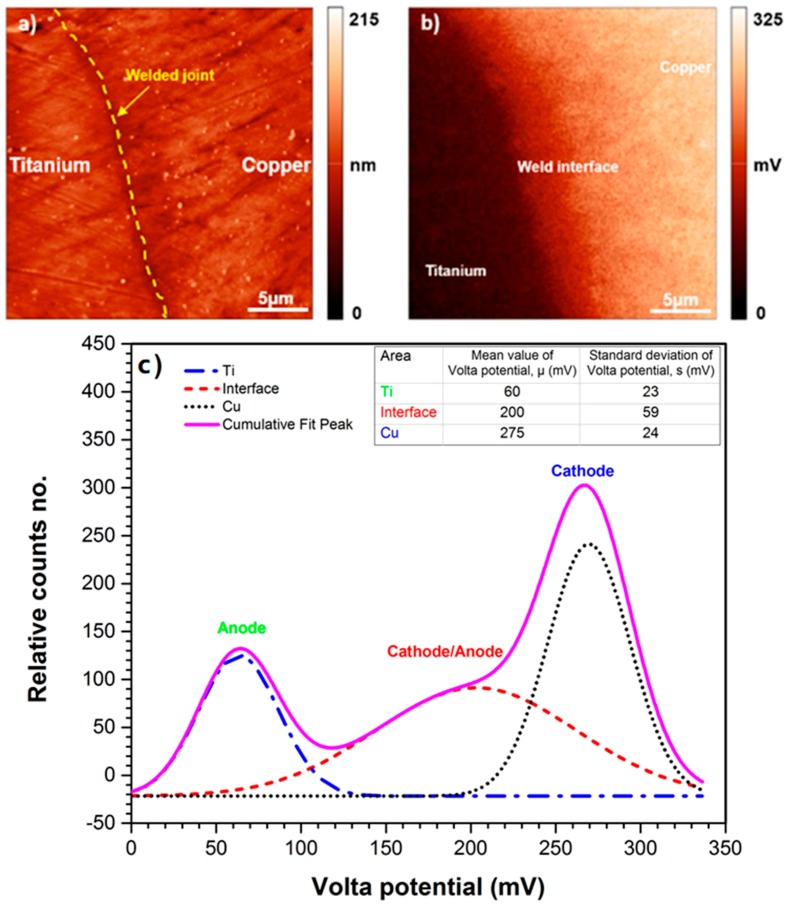
(**a**) AFM and (**b**) SKPFM image of Ti/Cu weld interface before immersion, (**c**) histogram analysis of Volta potential map obtained from (**b**); high Volta potential difference could be a reason for galvanic corrosion between Ti and Cu matrices.

The difference in the corrosion potential or nobility behavior of both metals in the Ti-Cu bimetal can be accurately explained with Scanning Kelvin Probe Force Microscopy (SKPFM) [54,55,56]. Figure 15a,b depicts the topographic AFM and SKPFM maps for an area of 25 µm × 25 µm of the polished cross-section at the weld interface between Cu and Ti. To interpret the Volta potential values analytically, in comparison with the conventional line profile method, a histogram plot of Volta potential map is calculated using the following multimodal Gaussian distribution equation [27], the details of which are provided elsewhere [25]:

(7) $$\;Y=\frac{1}{\sigma \sqrt{\frac{\pi }{2}}}exp\left[-\frac{2\left(x-\mu \right)}{\sigma^{2}}\right]\;$$

where *Y* represents the count number, *σ* is the standard deviation, *µ* is the mean potential value of Volta potential, and *x* is Volta potential values.

The difference between the mean values (i.e., Volta potential difference) is used to evaluate the galvanic driving force for corrosion initiation, while the standard deviation is employed for homogeneity evaluation of surface constituents [27]. According to histogram data, the weld interface has a higher standard deviation value (*σ* = 59 mV) than Ti and Cu regions, with values of 23 and 24 mV, respectively. This can be correlated with the heterogeneous distribution of melted zone adjacent to the weld interface, (inserted image in Figure 15c). Histogram analysis of the SKPFM image in Figure 15c shows a large potential gradient through the borderline between Ti and Cu metals with 215 mV driving force (ΔV = 275 − 60 mV). This large Volta potential difference drives the galvanic corrosion attacks between Ti and Cu metals [44].

**Figure 16:**
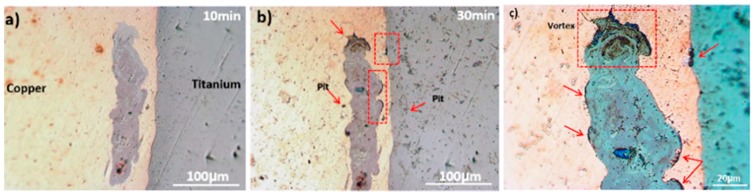
Optical micrograph of the galvanic corrosion initiation sites on welded interface and LMZ after immersion in 3.5 wt.% NaCl corrosive solution with different immersion times, (**a**) 10 min, and (**b**) 30 min. (**c**) is the greater magnification of LMZ in (**b**).

Accordingly, at the first moments of immersion, the Ti side and the melted zone are susceptible to corrosion attacks. To provide macroscopic evidence on the effect of Volta potential difference on the corrosion initiation, OM images are obtained from the joint interface in successive immersion times (10 min and 30 min), as shown in Figure 16. After 10 min immersion in corrosive medium (Figure 16a), the weld interface and melted zone, do not show any corrosion initiation sites in Ti-Cu bimetal. Figure 16b depicts that several corrosion initiation sites, marked with arrows, are formed after 30 min immersion in the corrosive solution. The corrosion initiation sites include weld interface, Cu-LMZ interface, and vortex zones in the LMZ (zoomed area in Figure 16c). The details of the nobility inversion upon continuation of the immersion of the sample to corrosive medium have been provided elsewhere [25].

## 4. Conclusions

The corrosion performance of a solid-state welded Ti-Cu bimetal was investigated during the initial h of immersion in 3.5% NaCl solution by means of AC/DC electrochemical measurements, OM, SEM, and SKPFM. Particular attention was paid to the effect of the microstructure on the initiation sites for corrosion attacks and its correlation to the Volta potential distribution along the joint interface and on the areas close to it. Based on the results provided in this study, the following points can be concluded:

Ti-Cu heterobimetallic complex is formed during the explosion welding of Ti and Cu, which includes the local melted zone mainly on the Cu side and the welded interface.

The higher heat conductivity of Cu and consequently its rapid solidification cause the formation of small local melted zones (1 µm approx.) in the welded interface.

The formation of Ti particles, local melted zones, and vortex regions on the Cu side result from the strong impact during the cladding process and the lower melting point of copper.

Ti particles on the Cu side, the local melted zone, and the welded interface are preferred potential sites for corrosion initiation.

Scanning Kelvin probe force microscope confirmed that for the as-received bimetal sample, the Cu side has a higher Volta potential value (about 215 mV) than the Ti side.

In the first moments of immersion, Ti acts as anode and Cu serves as cathode. However, after about 1 h of immersion, due to the growth of a passive film in contact with the aggressive medium, the difference in the potential between both metals begins to decrease until the net flow of electrons switches from the Cu side to the Ti side.

## Figures and Tables

**Table 1:** Electrochemical impedance spectroscopy parameters for Ti, Cu and weld interface electrodes in 3.5% NaCl at different immersion times.

Immersion Time	Surface	R_s_ (Ω·cm^2^)	R_f_ (kΩ·cm^2^)	CPE_f_ (µF·S^−1^·cm^−2^)	n_1_	R_ct_ (kΩ·cm^2^)	CPE_ct_ (µF·S^−1^·cm^−2^)	W (Ω·s^−0.5^)	n_2_
-	Titanium	25.2 ± 1	123.1 ± 2	45.3 ± 0.2	0.89 ± 00.5	-	-	-	-
	Copper	27.4 ± 2	0.51 ± 0.2	109.7 ± 1	0.72 ± 0.04	2.5 ± 0.6	240.8 ± 18	-	0.74 ± 0.01
	Interface	5.1 ± 0.5	0.55 ± 0.3	88.6 ± 0.3	0.78 ± 0.02	9.2 ± 0.4	291.6 ± 23	-	0.61 ± 0.04
1 h	Titanium	27.8 ± 3	4.7 ± 0.4	26.2 ± 3	0.97 ± 0.02	25.2 ± 2	12.9 ± 2	-	0.92 ± 0.03
	Copper	34.1 ± 2	0.53 ± 0.6	2.8 ± 0.7	0.89 ± 0.03	2.2 ± 0.4	188.5 ± 35	352 ± 3	0.51 ± 0.02
	Interface	42.1 ± 4	2.4 ± 0.2	7.5 ± 0.6	0.92 ± 0.05	9.1 ± 0.2	64.2 ± 2	4029 ± 30	0.52 ± 0.02
6 h	Titanium	28.3 ± 2	0.3 ± 0.1	32.5 ± 3	0.84 ± 0.04	5.2 ± 1	30.6 ± 2	-	0.89 ± 0.02
	Copper	13.4 ± 1	0.12 ± 0.1	75.8 ± 2	0.79 ± 0.03	0.7 ± 0.2	237.4 ± 50	150 ± 15	0.43 ± 0.01
	Interface	7.7 ± 2	1.2 ± 0.4	4.5 ± 2	0.92 ± 0.01	1.5 ± 0.3	8.4 ± 1	2626 ± 50	0.47 ± 0.05

**Table 2:** Potentiodynamic polarization parameters for Ti, Cu and weld interface electrodes in 3.5% NaCl at different immersion times.

Immersion Time	Surface	i_corr_ (µA·cm^−2^)	E_corr_ (mV vs. SCE)	ba (mV/decade)	bc (mV/decade)
0	Titanium	0.74 ± 0.3	−435.8 ± 2	297.1 ± 1	174.4 ± 1
	Copper	4.2 ± 0.2	−250.4 ± 1	56.3 ± 2	185.5 ± 3
	Interface	3.8 ± 0.4	−263.5 ± 1	90.2 ± 1	162.4 ± 2
1 h	Titanium	0.87 ± 0.4	−303.6 ± 2	301.8 ± 1	171.8 ± 2
	Copper	5.9 ± 1	−283.4 ± 2	214.2 ± 1	148.3 ± 1
	Interface	4.1 ± 1	−273.3 ± 2	150.8 ± 2	274.5 ± 2
6 h	Titanium	1.4 ± 0.5	−302.3 ± 2	197.2 ± 2	68.1 ± 2
	Copper	8.5 ± 0.3	−351.5 ± 2	104.8 ± 2	219.5 ± 1
	Interface	6.5 ± 0.5	−275.3 ± 2	138.7 ± 2	201.7 ± 2
